# Identification of a novel *LCA5* mutation in a Pakistani family with Leber congenital amaurosis and cataracts

**Published:** 2011-07-16

**Authors:** Adeel Ahmad, Shakeela Daud, Naseebullah Kakar, Gudrun Nürnberg, Peter Nürnberg, Masroor Ellahi Babar, Michaela Thoenes, Christian Kubisch, Jamil Ahmad, Hanno Jörn Bolz

**Affiliations:** 1 Department of Biotechnology and Informatics, BUITEMS, Quetta, Pakistan; 2 Institute of Biochemistry and Biotechnology, UVAS, Lahore, Pakistan; 3 National Centre of Excellence in Molecular Biology, University of the Punjab, Lahore, Pakistan; 4 Cologne Center for Genomics and Institute for Genetics, University of Cologne, Cologne, Germany; 5 Cologne Excellence Cluster on Cellular Stress Responses in Aging-Associated Diseases, University of Cologne, Cologne, Germany; 6 Institute of Human Genetics, University Hospital of Cologne, Cologne, Germany; 7 Institute of Human Genetics, University of Ulm, Germany; 8 Center for Human Genetics, Bioscientia, Ingelheim, Germany

## Abstract

**Purpose:**

To determine the cause of Leber congenital amaurosis (LCA) and developmental cataracts in a consanguineous Pakistani family.

**Methods:**

The diagnosis was established in all affected individuals of a Pakistani LCA family by medical history, funduscopy, and standard ERG. We performed genome-wide linkage analysis for mapping the disease locus in this family.

**Results:**

Congenitally severely reduced visual acuity and nystagmus were reported for all patients who, in the later phase of the disease, also developed cataracts. LCA in the family cosegregated with homozygosity for a single nucleotide polymorphism (SNP) haplotype on chromosome 6p14.1. The respective candidate region contained Leber congenital amaurosis 5 (*LCA5*), a gene previously reported to underlie LCA. We subsequently identified a novel truncating mutation in exon 4 of *LCA5*, c.642delC, in homozygous state in all affected persons of the family.

**Conclusions:**

We report a novel *LCA5* mutation causing LCA in a Pakistani family. Developmental cataracts were present in two of the four patients, raising the possibility that *LCA5* mutations may predispose to this additional ocular pathology.

## Introduction

Leber congenital amaurosis (LCA, OMIM 204000) accounts for at least 5% of all retinal dystrophies and approximately 20% of children attending schools for the blind. LCA is the most severe retinal dystrophy causing blindness or severe visual impairment before the age of 1 year. Inheritance is autosomal recessive in most cases. Clinically, LCA is characterized by the presence of four key features, namely severe and early visual loss (usually around the age of 6 weeks), sensory nystagmus, amaurotic pupils, and minimal or absent responses in the electroretinogram (ERG). LCA can be observed as part of syndromes such as Joubert syndrome. To date, 15 genes associated with LCA have been identified [1]. These genes are involved in various genetic pathways, including retina development (crumbs homolog-1 [*CRB1*] and cone-rod homeobox-containing gene [*CRX*]*),* phototransduction (guanylate cyclase 2D [*GUCY2D*] and aryl hydrocarbon receptor interacting protein-like 1 [*AIPL1*]), vitamin A metabolism (retinal pigment epithelium-specific protein 65 kDa [*RPE65*]; lecithin retinol acyltransferase [*LRAT*], and retinol dehydrogenase 12 [*RDH12*]), ciliary formation and function (tubby like protein 1, [*TULP1*]; retinitis pigmentosa GTPase regulator interacting protein 1, [*RPGRIP1*]; centrosomal protein 290 kDa [*CEP290*], and Leber congenital amaurosis 5 [*LCA5*]), and RPE phagocytosis (c-mer proto-oncogene tyrosine kinase [*MERTK*]). The function of *RD3* remains to be elucidated. In rare cases, certain mutations in *CRX* and inosine 5′-monophosphate dehydrogenase 1 (*IMPDH1*), which is involved in guanine nucleotide synthesis, have been shown to cause dominant LCA. Recently, another gene, *SPATA7* was identified as the *LCA3* gene [2]. Mutations in the known LCA genes account for ~70% of non-syndromic LCA cases. *LCA5* mutations probably account for less than 3% of all cases.

Although many persons with LCA may have normal or near normal fundus appearance as infants, pigmentary retinopathy as in retinitis pigmentosa may develop at later stages (RP). Consistent with this observation, four known LCA disease genes, including *CRX*, *CRB1*, *RPE65*, and *TULP1*, have also been linked to RP. Mutations in LCA disease genes may lead to diverse phenotypes, e.g., cone rod dystrophy (*CRX*, *AIPL1*, *GUCY2D*, *RPE65*, and *RPGRIP1*), retinal dystrophy (*RDH12*), and Bardet-Biedl, Joubert or Meckel syndrome (*CEP290*). Therefore, the study of LCA provides potential insight into other retinal dystrophies and genetic syndromes [1].

**Figure 1:**
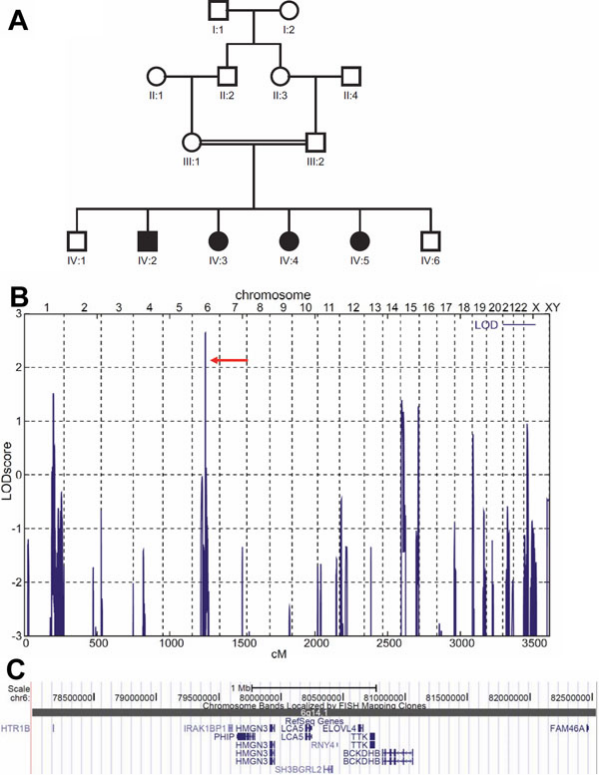
Genetic analysis of LCA family BUIT-LA01. **A**: Family pedigree. Circles, females; squares, males. Black symbols, affected. Double horizontal line indicates consanguinity. The sample of IV:5 was not subjected to genomewide SNP mapping because this sample was initially not available. **B**: Graphical view of LOD score calculations of genomewide SNP mapping in the BUIT-LA01 family. LOD scores calculated with ALLEGRO are given along the y-axis relative to genomic position in cM (centi Morgan) on the x-axis. Note the highest peak in the region on chromosome 6 (LOD=2.66). **C**: Corresponding disease gene locus on chromosome 6q14.1 (4.5 Mb region, only 11 annotated genes; UCSC genome browser).

Here, we report a consanguineous family from Pakistan with four affected individuals diagnosed with autosomal recessive LCA (Figure 1A). Genomewide linkage analysis mapped the disease region to chromosome 6p14.1 which contained *LCA5* (Figure 1B,C). Mutational analysis identified a homozygous novel frameshift mutation (c.642delC) in exon 4 of *LCA5* in all patients.

## Methods

### Family enrollment and clinical evaluation

A consanguineous family, BUIT-LA01, with four individuals affected by LCA was enrolled from a remote area in Pakistan (Figure 1A). The diagnosis was established in all patients (IV:2, IV:3, IV:4, and IV:5) by medical history, decreased visual acuity since birth, nystagmus, funduscopy and by standard ERG. Clinical history was obtained from participating members to rule out obvious environmental causes of vision impairment. There was no history of deafness, mental retardation, or any other associated disease related to the patients or other family members (IV:1, IV:6). The study was approved by the institutional review board (IRB) at the Department of Biotechnology and Informatics, BUITEMS, Quetta, Pakistan, and the institutional review board of the Ethics Committee of the University Hospital of Cologne, Germany. It was performed in adherence to the tenets of the declaration of Helsinki. After detailed explanation to the family members about the background of the study, written consent was obtained from all participants. Venous blood samples (5 ml) were obtained for DNA extraction and genomic DNA was isolated following standard protocols [3]. Standard electroretinograms (LKC Technologies) were measured under scotopic and photopic conditions in the elder affected patient (IV:3) according to the standard of the International Society for Clinical Electrophysiology of Vision beginning after 20 min of dark adaptation [4]. Pupils were fully dilated using phenylephrine HCL (10%) and tropicamide (1%). To study the retinal features, funduscopy was performed on two of the elder affected individuals (IV:3 and IV:5) of the family.

### Linkage analysis

Samples from the parents and all children except IV:5 (this sample was collected at a later point of time) were subjected to a whole genome scan, using an Affymetrix GeneChip Human Mapping 10K Array, version 2.0 (Affymetrix, Santa Clara, CA). GRR and PedCheck were used to verify relationships and to identify Mendelian errors [5,6]. Non-parametric linkage analysis was performed with MERLIN [7]. Parametric linkage and haplotype analysis were performed using ALLEGRO [8], assuming autosomal recessive inheritance, full penetrance and a disease gene frequency of 0.0001. All data handling was performed using the graphical user interface ALOHOMORA [9]. Graphic output of haplotypes was generated with HaploPainter [10].

### Mutational analysis

The seven coding exons and adjacent intronic sequences of the *LCA5* gene (NM_181714) were amplified and sequenced (Table 1). PCR products were amplified using 100 ng of genomic DNA in a 25 µl reaction mixture containing 10 pmol of forward and reverse primers, 0.2 mM dNTP, 10 mM Tris-HCl, 50 mM KCl, 1.5 mM MgCl_2_, and 0.5 units of Taq polymerase (Invitrogen Corp., Carlsbad, CA). After initial denaturation at 95 °C for 4 min, 30 cycles were performed, which consisted of 95 °C for 1 min, 55–62 °C (depending on the fragment) for 1 min, and 72 °C for 1 min, with a final extension step of 72 °C for 10 min for all exons. PCR products were digested with exonuclease I and shrimp alkaline phosphatase (Fermentas Life Sciences, Glen Burnie, MD) and sequenced bi-directionally using BigDye Terminator v.3.1 kit (Applied Biosystems, Darmstadt, Germany). DNA mutation nomenclature of identified mutation was based on cDNA sequence of the longest isoform of *LCA5* with +1 corresponding to the A of the ATG translation initiation codon (codon 1) in the respective reference sequence (GenBank NM_181714). For the description of sequence variation, we followed the recommendations of the Human Genome Variation Society (HGVS). Samples from 50 ethnically matched healthy controls from Pakistan were investigated by direct sequencing for the presence of the mutation.

**Table 1:** Primers used for PCR amplification and direct sequencing of *LCA5* coding exons and adjacent intronic regions.

**Exon**	**Primer sequence**	**tm (°C)**	**cds (bp)**	**Product size (bp)**
3F	TGTGGAGAAAATAGATTGCACAG	59.28		
3R	CCTATAAAACGTAAATCAGCCCAC	60.14	190	465
4F	AGAATAATTCCGTATAAACTATTGGG	57.42		
4R	TTTTCCCAAAATGACTATGATCC	59.22	530	996
5F	TGTACATGAATACTATGCCCAGTC	58.10		
5R	TTATACCAACAAACCTTTTCTAAGTG	57.26	138	437
6F	CAAAAGGAAGCTGAACCAGG	59.85		
6R	CAGCGTCACTTCAGGGG	58.83	97	420
7F	CCAAGCTGAGCAAAACATGC	61.88		
7R	TTAGGTATATCTCCTAAAAGCCAAAG	56.64	143	538
8F	TTCAAGGAGTGATAACTTGTGATTTAC	59.12		
8R	TAAGCCATCCCCTACCACTG	59.95	133	437
9F	TCTTTTCTCACTTGATTTATAATACCC	57.59		
9R	TTGGCAAACTATCTATGTGGTG	57.72	863	1167

## Results

**Figure 2:**
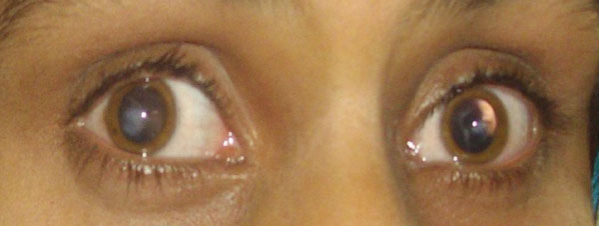
Photograph of patient IV:3 showing bilateral cataracts (radial spoke-shaped) and keratoconus.

**Figure 3:**
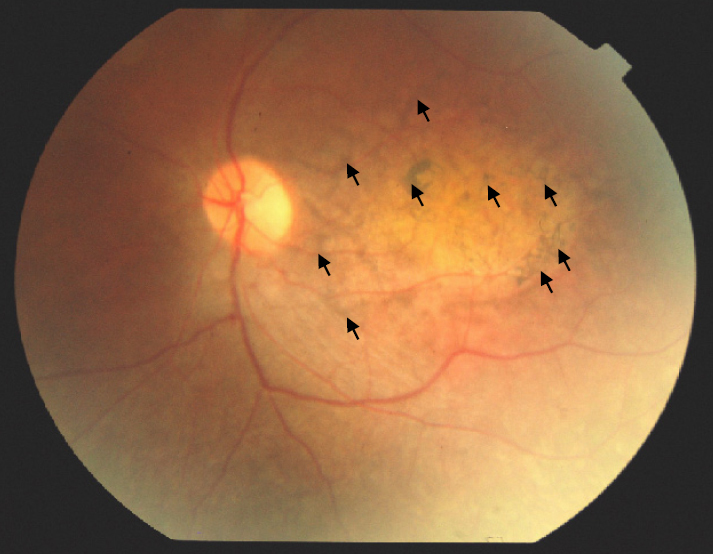
Fundus photograph of patient (IV:4) with waxy pale disc appearance and pigmentary changes (arrows) in the macular region and the posterior pole of the retina.

**Figure 4:**
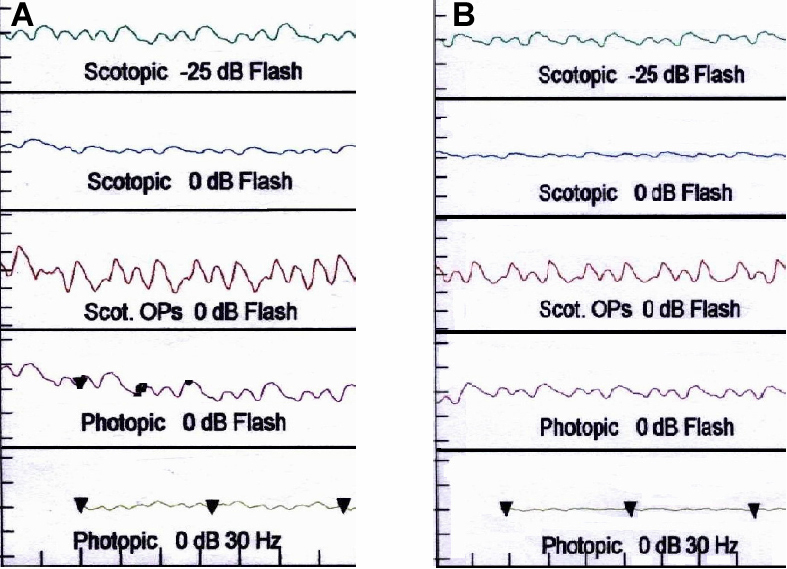
Electroretinogram (ERG) profile of IV:3. **A**: Right eye of IV:3. **B**: Left eye of IV:3.

All affected individuals of BUIT-LA01 displayed severe visual impairment since birth, bilateral keratoconus and nystagmus (Figure 1A). Slit lamp examination showed radial spoke-shaped cataracts with positive oculodigital reflex in the two older patients who were 13 and 15 years old (Figure 1A; IV:3, IV:5; and Figure 2). Cataracts were (until now) not present in the two younger patients (IV:2, IV:4; Figure 1A) who are 7 years and 9 years old. The fundus features showed moderately attenuated vessels, waxy pale disc appearance, pigmentary changes especially at macular areas in both eyes, and some bony spicule-like pigments clumping in the peripheral region and posterior pole of the retina (Figure 3). ERGs showed normal implicit time with reduced rods and cones function as a whole and markedly reduced response from rods. Results of scotopic ERGs were markedly abnormal, with the ISCEV standard, flash conducted by −25 dB, and the rod b-wave amplitude was severely reduced with a normal implicit time. Under photopic conditions, the b-wave amplitude was moderately reduced while photopic 30-Hz flicker stimulus revealed decreased amplitude with normal implicit time in both eyes (Figure 4 and Table 2).

**Table 2:** Standard flash ERG values in affected individual (IV:3) and normal values.

**Patient (IV:3)**	**Scotopic (−25 dB)**	**Scotopic (0 dB)**	**Photopic (0 dB)**	**Photopic/ flicker (30 Hz)**
Right Eye	46.5	68.7	64.6	3.6
Left Eye	126	89.1	76.1	4.8
Normal control	167	371	94	62
Normal values	185	419	102	70

Genomewide linkage analysis revealed a maximum parametric LOD (logarithm of the odds)  score of 2.66 for a 4.5 Mb region on chromosome 6q14.1, flanked by single nucleotide polymorphisms (SNPs) rs2153886 and rs72968, that showed homozygosity by descent in all four affected siblings (Figure 1B). This critical interval contained 11 genes, including *LCA5*, the gene encoding lebercilin, a protein of the photoreceptor connecting cilium. Mutational analysis identified a novel frameshift mutation (c.642delC) in exon 4 of *LCA5* in homozygous state in all patients from family BUIT-LA01. This variant should either lead to a subsequent truncation of the protein (225 instead of 697 residues in the wildtype), or an unstable mRNA molecule that is rapidly degraded. The mutation was absent in 50 unrelated control individuals from Pakistan.

## Discussion

Mutations in the ciliary protein lebercilin, encoded by *LCA5*, have been shown to cause LCA. Recent data have shown that lebercilin functions in selective protein transport through the photoreceptor’s connecting cilium, suggesting defective intraflagellar transport as underlying disease mechanism [11]. To date, 10 mutations in the respective gene, *LCA5*, have been reported ([12–16], HGMD). Here, we report the identification of a novel mutation (c.642delC) in exon 4 of *LCA5* in a consanguineous family from Pakistan. The affected individuals are between 7 and 15 years old and all presented with typical signs of LCA at birth. There were no signs for a syndromic entity at the age of examination (e.g., renal failure, neurologic symptoms, mental retardation, dysmorphic features). The phenotype of the family described herein is consistent with the phenotype described previously in *LCA5* patients [13,15–17]. An additional phenotypic feature observed in our family were radial spoke-shaped developmental cataracts (illustrated in Figure 2 for individual IV:3) in the two older patients (13 years and 15 years old), while cataracts were not present in the two younger patients (IV:2, IV:4; Figure 1A) who were at 7 years and 9 years of age, respectively. Although LCA is a congenital abnormality, a previous report of a Pakistani *LCA5* family suggested further progression of the phenotype, but not including cataracts [18]. The patients of that already-reported Pakistani *LCA5* family had phenotypes that are different from that of the original *LCA5* family of Swiss descent [17]. Visual acuity was already poor at birth (reduced to light perception in the second decade), and the disease progressed with pigmentary anomalies in the peripheral retina and increasing atrophy in the macular region [18]. The phenotype of the patients from three families from Maghreb resembled to that of the previously reported *LCA5* families. There were no signs of cataracts, although the patient were between 17 and 40 years old [13]. In a recent study on 14 LCA families (3 families linked to *LCA5*) from Northern Pakistan, the prevalence of cataracts in *LCA5* was estimated to be 8%. In that study, cataracts represent a late-onset feature of *LCA5* and did not manifest before the second decade of life [15]. Our study suggests that cataracts may also be an early feature in *LCA5*-linked LCA. Alternatively, given the parental consanguinity in our *LCA5* family, it cannot be excluded that the manifestation of cataracts in two patients reflects homozygosity for a mutation in an unlinked (cataract) gene. We have previously described such constellations with two overlapping recessive conditions, mimicking a single, syndromic disorder [19,20]. Furthermore, LCA-associated cataracts have been postulated to result from a combination of genetic and environmental factors [1].

In summary, we describe a Pakistani family with Leber congenital amaurosis linked to the *LCA5* locus, and the identification of a novel frameshift mutation confirms *LCA5* as contributor to congenital retinal degeneration. The finding of early-onset cataract in two patients from our family extends the *LCA5* phenotype spectrum. Finally, our study confirms the potential of homozygosity mapping in recessive retinal degeneration families from inbred populations.

## 

**Table:** In the Table, tm indicates melting temperature; cds indicates coding sequence; bp indicates product size in basepairs.
